# Impact of Matrix Metalloproteinases 11 Gene Variants on Urothelial Cell Carcinoma Development and Clinical Characteristics

**DOI:** 10.3390/ijerph17020475

**Published:** 2020-01-11

**Authors:** Chien-Chang Li, Ming-Ju Hsieh, Shian-Shiang Wang, Sheng-Chun Hung, Chia-Yen Lin, Chi-Wen Kuo, Shun-Fa Yang, Ying-Erh Chou

**Affiliations:** 1 Department of Urology, Jen Ai Hospital, Taichung 400, Taiwan; lccsur2003@pchome.com.tw; 2 Institute of Medicine, Chung Shan Medical University, Taichung 402, Taiwan; 170780@cch.org.tw (M.-J.H.); sswdoc@vghtc.gov.tw (S.-S.W.); weshong1118@gmail.com (S.-C.H.); lcyhank.tw@gmail.com (C.-Y.L.); ysf@csmu.edu.tw (S.-F.Y.); 3 Cancer Research Center, Changhua Christian Hospital, Changhua 500, Taiwan; 4 Graduate Institute of Biomedical Sciences, China Medical University, Taichung 404, Taiwan; 5 School of Medicine, Chung Shan Medical University, Taichung 402, Taiwan; 6 Division of Urology, Department of Surgery, Taichung Veterans General Hospital, Taichung 407, Taiwan; 7 Department of Applied Chemistry, National Chi Nan University, Nantou 545, Taiwan; 8 Department of Pharmacy, Jen-Ai Hospital, Taichung 400, Taiwan; 6662010@gmail.com; 9 School of Pharmacy, College of Pharmacy, China Medical University, Taichung 404, Taiwan; 10 Basic Medical Education Center, Central Taiwan University of Science and Technology, Taichung 406, Taiwan; 11 Department of Health Policy and Management, Chung Shan Medical University, Taichung 402, Taiwan; 12 Department of Medical Research, Chung Shan Medical University Hospital, Taichung 402, Taiwan

**Keywords:** urothelial cell carcinoma, MMP11, polymorphism

## Abstract

Urothelial cell carcinoma (UCC) is one of the lethal causes of cancer mortality of the genitourinary tract. Carcinogenic epidemiological risk factors exposure and age over 65 years old are associated with UCC risk. Matrix metalloproteinase 11 (MMP11) was suggested as a tumor marker of metastasis and predictor of poor survival in urothelial carcinomas. In this study, we focused on the associations of MMP11 single-nucleotide polymorphisms (SNPs) to UCC susceptibility, clinicopathological characteristics, and prognosis. In this study, real-time polymerase chain reaction was used to analyze five SNPs of MMP11 rs738791, rs2267029, rs738792, rs28382575, and rs131451 in 431 patients with UCC and 650 cancer-free controls. The MMP11 rs28382575 polymorphic “CT” genotype were susceptible to UCC (AOR = 2.045, 95% CI = 1.088 − 3.843; *p* = 0.026). For MMP11 rs131451, a significant association was found in 166 UCC patients among age ≤ 65 years old who carried MMP11 rs131451 polymorphic “CC” genotype, which is associated with lower risk to develop later tumor T status (T1-T4) (OR = 0.375, 95% CI = 0.159 − 0.887; *p* = 0.026) compared with the (CT + TT) genotype. Furthermore, patients of UCC with rs738792 polymorphic “CC” genotype were observed to have higher free of relapse (FS) (*p* = 0.035), disease specific survival rate (*p* = 0.037), and overall survival rate (*p* = 0.009) compared with the rs738792 (CT + CC) genotype. In conclusion, our results demonstrated that the MMP11 SNPs are associated with UCC susceptibility, clinical status, and disease survival. The MMP11 polymorphisms may have potential to predict UCC susceptibility and prognosis.

## 1. Introduction

Urothelial cell carcinoma (UCC) of the urinary bladder cancer (UBC) is responsible for approximately 3.0% of all newly diagnosed cancer and 2.1% of all cancer deaths [1]. In Taiwan, urothelial carcinoma is the most common type of bladder cancer which accounts for 98.65% cases, with a male-to-female predominance ratio of 2.6:1 [2]. Increasing age is one of the major risk factors for bladder cancer [3]. Carcinogenic risk factor such as smoking and exposure to some industrial chemicals also result in increased the risk of urinary bladder carcinoma [3,4,5,6,7,8].

Matrix metalloproteinase 11 (MMP11), or stromelysin-3, was first identified in the stromal cells of breast carcinoma [9,10]. MMP11 is associated with tissue remodeling during embryogenesis, extracellular matrix (ECM) remodeling and/or degradation, epithelium growth, metamorphosis, and wound healing [11,12,13]. The overexpression of MMP11 has been observed in various types of human cancers and was suggested to be associated with cancer aggressiveness and invasiveness [9,11,14,15,16,17,18,19,20,21,22]. Knockdown of MMP11 expression was observed to inhibit the proliferation and invasion of human gastric adenocarcinoma (GAC) cells, and it was suggested that the down-regulated Insulin-like growth factor-1 (IGF-1) signaling pathway might be responsible for this phenomenon [23]. Recent study has suggested that the MMP11 may play a role as a marker of metastasis and predictor of poor survival in urothelial carcinomas [11].

The single nucleotide polymorphisms (SNPs) of the MMP members have been suggested to be correlated with bladder cancer risk and invasiveness [24,25,26,27,28,29,30,31,32,33,34]. However, information regarding the MMP11 gene polymorphisms to UCC of the urinary bladder remained limited and uninvestigated. In this study, we use real-time polymerase chain reaction to analyze five SNPs of MMP11 rs738791, rs2267029, rs738792, rs28382575, and rs131451 in 431 patients with UCC and 650 cancer-free controls, and try to elucidate their correlations in UCC development and clinicopathological characteristics.

## 2. Materials and Methods

### 2.1. Subject Selection

In the current study, 431 UCC patients including 272 men and 159 women (mean age = 68.6 ± 11.8 years) were enrolled as the study group. These patients were diagnosed and follow-up during 2010 to 2013 at the Taichung Veterans General Hospital in Taichung, Taiwan. For the control group, 650 age and gender matched healthy control including 240 men and 410 women (mean age = 64.1 ± 3.1 years) that entered the hospital physical examination were recruited in our study. The study was approved by the Institutional Review Board of Taichung Veterans General Hospital (IRB no. CF11094). At the time of diagnosis, the clinical staging of the UCC patients were staged according to the tumor/node/metastasis staging system of the American Joint Committee on Cancer (AJCC) [35,36]. Individuals of the control groups who involved in this study had neither self-reported history of cancer of any sites. For personal characteristics and information collection, we use interviewer-administered questionnaires containing questions with demographic characteristics to acquire the related information from study subjects for the study. Informed written consent was obtained from each individual before the initiation of the study.

### 2.2. Sample Preparation and DNA Extraction

For genomic DNA extraction, the peripheral blood specimens were collected from UCC patients and normal controls enrolled in our study. The extracted whole blood samples were stored in EDTA containing tubes, and then centrifuged at 3000 rpm for 10 min. For DNA collection, the genomic DNA extraction assay was performed with QIAamp DNA blood mini kits following the instructions of the manufacturer’s protocol. Extracted buffy coats from the whole blood specimens were used for the DNA extraction. The final eluted DNA was dissolved in Tris-EDTA (TE) buffer, and the final extracted DNA was applied as DNA template in the next research process of the polymerase chain reactions (PCRs).

### 2.3. Selection of MMP11 SNPs

A total of five SNPs rs738791, rs2267029, rs738792, rs28382575, and rs131451 in MMP11 were selected from the International HapMap Project database for this study [37]. The MMP11 rs738791 was selected because this SNP was suggested to be associated with greater risk of hepatocellular carcinoma (HCC) and cervical invasive cancer [38,39]. The MMP11 SNP rs738792 was selected because carriers of at least one C allele (C/T + C/C genotype) were suggested to be more likely to progress to Child-Pugh B or C grade in patients of hepatocellular carcinoma (HCC) [38], and it was found that the patients of oral squamous cell carcinoma (OSCC) who possess at least one polymorphic C allele of MMP11 rs738792 were associated with increased incidence of lymph node metastasis compared with the homozygous T/T genotype [40]. The MMP11 SNP rs28382575 was selected because it was found that individuals with at least one C allele (C/T + C/C genotype) were associated with higher risk to develop, large tumors, lymph node metastasis, or stage III/IV disease in HCC [38].

### 2.4. MMP11 SNPs Genotyping

Assessment of allelic discrimination for the MMP11 rs738791 (assay IDs: C___2448099_30), rs2267029 (assay IDs: C__15871447_20), rs738792 (assay IDs: C___2213764_20), rs28382575 (assay IDs: C__61238655_10), and rs131451 (assay IDs: C___2213679_30) SNP was performed with an ABI StepOne Software v2.3 Real-Time PCR System, and the TaqMan assay was used for genotyping. The final data were collected and further analyzed with SDS 7000 series software (Applied Biosystems, Foster City, CA, USA).

### 2.5. Statistical Analysis

To compare the age, gender, tobacco consumption, stage (non-muscle invasive tumor and muscle invasive tumor), tumor T status, lymph node status, metastasis, histopathologic grading between the healthy controls and patients with UCC, student’s t test or Chi-squared test was used between these two groups. *p* < 0.05 was considered a significant does exist. To estimate the odds ratio (OR) with their 95% confidence intervals (CIs) of the association between the genotype frequencies and UCC risk and the clinical pathological characteristics, logistic regression models were used for data analysis. To estimate the hazard ratio (HR) and adjusted hazard ratio (AHR) with their 95% confidence intervals of the association between the risk of death and genotypic distributions of *MMP11* gene polymorphisms, Cox proportional hazards model was performed with adjustment for confounding factors including age, gender and tobacco consumption. All of the data involved in this study were analyzed with SAS statistical software (Version 9.1, 2005; SAS Institute, Cary, NC, USA).

## 3. Results

The distribution of demographical characteristics in 650 controls and 431 patients with UCC were demonstrated in Table 1. After we analyzed these demographical characteristics, we observed that 32.6% (212/650) of the controls and 30.4% (131/431) of the patients with UCC consumed tobacco. A significant distributional difference was observed for age (*p* < 0.001) between the controls and patients with UCC.

The genotyping and allele frequency of MMP11 SNPs in the patients with UCC and healthy controls are presented in Table 2. The highest distribution frequencies in the controls and patients with UCC of MMP11 genetic polymorphisms rs2267029, rs738792, rs28382575, and rs131451 were homozygous for GG, homozygous for TT, homozygous for TT, and heterozygous for CT, respectively. However, the highest distribution frequencies of the MMP11 rs738791 polymorphisms in controls were homozygous for GG and heterozygous for CT in UCC patients, respectively. After adjustment for the effects of age, gender, and tobacco, a statistical significant association was found for the patients with UCC and MMP11 rs28382575 polymorphisms and those with the wild-type (WT) gene. However, no significant differences were observed for the patients with UCC among the rs738791, rs2267029, rs738792, and rs131451 polymorphisms of the MMP11 gene and those with the wild-type (WT) gene (Table 2).

To clarify the role of MMP11 genetic polymorphisms in UCC status in relation to clinical stage, tumor T status, lymph node status, metastasis and histopathologic grading, the distribution frequency of the clinical status and MMP11 genotype frequencies in 431 UCC patients was estimated. However, the MMP11 rs738791, rs2267029, rs738792, and rs28382575 genetic polymorphisms showed no significant association with clinicopathologic status (data not shown). Intriguingly, although the MMP11 SNP rs131451 genotypic variants showed no statistical significant association to the clinical status in 431 UCC patients (Table 3), we found that carriers with the MMP11 rs131451 “CC” genotype had a lower risk (The odds ratio (OR) = 0.375, 95% confidence intervals (CI) = 0.159 − 0.887) to develop advanced-stage of tumor t status in 166 UCC patients among age ≤ 65 years old compared with the MMP11 rs131451 “CT + TT” genotype (*p* = 0.026, Table 4).

**Figure 1:**
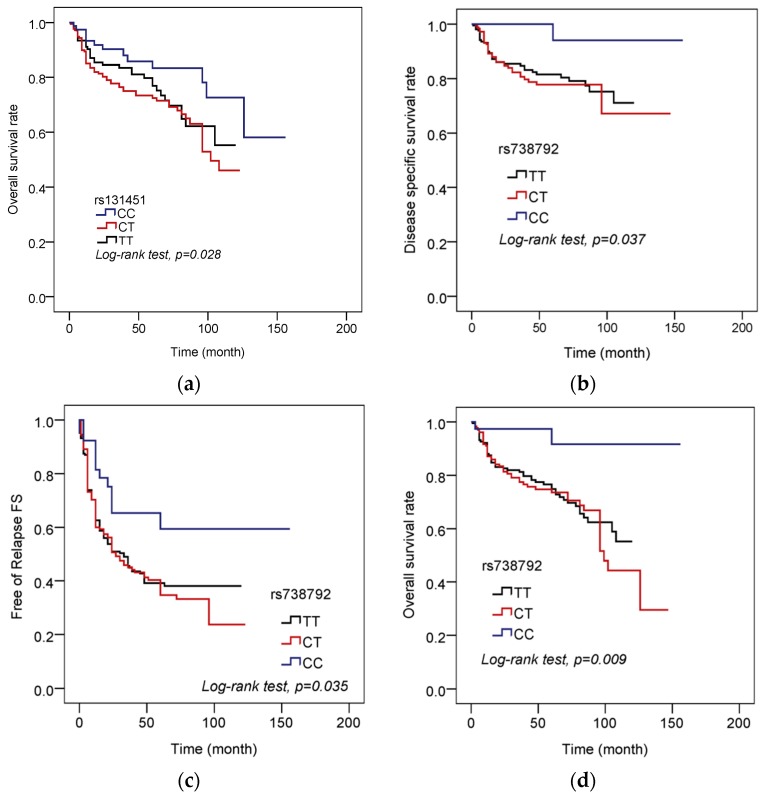
Distribution frequency of the overall survival rate, free of relapse and MMP11 polymorphism in 431 UCC patients. (**a**) Overall survival rate and MMP11 rs131451 genotype frequencies. (**b**) Disease specific survival rate and MMP11 rs738792 genotype frequencies. (**c**) Free of relapse (RS) and MMP11 rs738792 genotype frequencies. (**d**) Overall survival rate and MMP11 rs738792 genotype frequencies.

We further analyzed the associations of overall survival rate, free of relapse, and disease specific survival rate between MMP11 SNPs and the UCC patients. Individuals who carried MMP11 rs131451 “CC” genotype showed higher overall survival rate compared with the “CT + TT” genetic variants in patients with UCC (Figure 1a; *p* = 0.028). For MMP11 SNPs rs738792, patients of UCC with rs738792 CC genotype were observed to have higher free of relapse (FS) (Figure 1b; *p* = 0.035), disease specific survival rate (Figure 1c; *p* = 0.037), and higher overall survival rate (Figure 1d; *p* = 0.009) compared with the rs738792 “CT + TT” polymorphisms.

To estimate the risk of death on genotypic distributions of *MMP11* gene polymorphisms (rs131451, rs738792), the hazard ratio (HR) and adjusted hazard ratio (AHR) with their 95% confidence intervals were evaluated by Cox proportional hazards model among 430 UCC patients (one unmatched case was excluded). The results showed that patients with the MMP11 rs131451 “CT + TT” genotype had a higher risk (The AHR = 2.05, 95% CI = 1.12−3.754) for cancer mortality compared with the “CC” wild-type carriers. For MMP11 rs738792, patients with the “CT + TT” genotype had a higher risk (AHR = 6.562, 95% CI = 1.612−26.704) for cancer death compared with the “CC” wild-type carriers (Table 5).

## 4. Discussion

In our study, we demonstrated the correlations of MMP11 SNPs among UCC susceptibility, clinical status, disease prognosis and survival. Tobacco consumption and increasing age were suggested as major risk factors for bladder cancer carcinogenesis [3,7], and it was observed and suggested that the urothelial carcinoma is a male predominant disease [2]. In the current study, we found that there is no statistical significant in gender (*p* = 0.991) and tobacco consumption (*p* = 0.442) between the controls and the UCC patients (Table 1), suggesting that the impact of sex difference and cigarette smoking to bladder cancer carcinogenesis may be limited in those individuals involved in our study (Table 1). However, a statistical significant was found in age between the study group and the controls (*p* < 0.001; Table 1). Therefore, it seemed that the age >65 was the dominant risk factor for UCC carcinogenesis for these study subjects involved in our study.

We further analyzed the genotype distributions of MMP11 gene polymorphisms in controls and patients with UCC. The result exhibited that a statistical significant was found in those individuals who carried MMP11 rs28382575 “CT + CC” compared with the wild-type “TT” polymorphisms (*p* = 0.026, AOR: 2.045, 95% CI: 1.088–3.843; Table 2). However, no significant association was found in MMP11 SNPs rs738791, rs2267029, rs738792, and rs131451 between the UCC patients and the control group (Table 2). Previous studies have linked the MMP11 polymorphisms with cancer risk and tumor development, and it seemed that the impact of MMP11 SNPs to cancer susceptibility varied in different cancers [38,40,41]. In a study focused on lung cancer of Taiwanese patients, it was suggested that the genotypes of MMP11 might play a minor role in determining lung cancer risk since no significant association was found between the MMP11 rs738791, rs2267029, rs738792 or rs28382575 and lung cancer risk, and no joint effect of MMP11 genotypes was found among ever smokers or non-smokers [41]. For uterine cervical cancer in Taiwanese women, although the MMP11 gene CT/TT genotype of rs738791 tended to increase the risk of developing stage II disease and deep stromal invasion more than 10 mm with the CC genotype as a reference in these patients, it became insignificant after adjustments were applied, and no impact of the MMP11 SNPs on these patients was suggested [39]. Intriguingly, for hepatocellular carcinoma (HCC), it was suggested that carriers of the rs738791 CT + TT genetic variants were at higher risk of HCC compared with the wild-type (CC) carriers [38]. Furthermore, carriers who possess at least one C allele (C/T + C/C genotype) of the MMP11 SNP rs738792 were more likely to develop Child-Pugh B or C grade, while individuals who carried (C/T + C/C genotype) at the MMP11 SNP rs28382575 were observed to have higher risk to develop large tumors, lymph node metastasis or stage III/IV disease [38]. Compared with these results, our study indicated that the MMP11 rs28382575 CT + CC polymorphisms was associated with UCC susceptibility (*p* = 0.026; Table 2). In contrast, the MMP11 rs28382575 polymorphic variants showed no significant association to cancer susceptibility, neither in lung cancer nor uterine cervical cancer in women [39,41]. Further, no significant association was found for MMP11 rs738792 polymorphisms to UCC patients in our study, whereas the MMP11 rs738792 polymorphic variants were associated with cancer progression and development in HCC [38]. These results demonstrated the varieties of MMP11 polymorphisms to cancer development and tumor progression in different cancers. However, the detail mechanisms and regulations of these MMP11 SNPs remained incompletely understood.

We further analyzed the correlations between the MMP11 polymorphisms and the distribution frequency of the clinical status in 431 UCC patients. However, no significant association was found between the MMP11 SNPs rs738791, rs2267029, rs738792, and rs28382575 polymorphic variants and clinical status of the UCC patients (data not shown). Intriguingly, among these MMP11 SNPs selected in our study, although the MMP11 rs131451 polymorphisms “CC” genotype were not associated with the clinical status in 431 UCC patients involved in our study (Table 3), we found that the MMP11 rs131451 “CC” polymorphic variants were associated with lower risk to develop later tumor T status compared with the “CT + TT” genotype in 166 UCC patients among age ≤ 65 years old (*p* = 0.026, OR: 0.375, 95% CI: 0.159–0.887; Table 4). Although the effects of MMP11 rs131451 and the correlations between aging and MMP11 SNPs remained limited, it was suggested that lower expression of MMP11 was associated with better overall survival of bladder cancer patients [42], suggesting that the MMP11 rs131451 “CC” variants might be associated with lower level of MMP11 expression and protective effect among UCC patients whose age ≤65 years (Table 4). Further, we further analyzed the correlations between the MMP11 SNPs and the UCC prognosis and survival. Surprisingly, we discovered that the UCC patients with rs131451 “CC” genotype were associated with better overall survival rate (Figure 1a), and patients who possess the rs738792 “CC” genotype were associated with better free of relapse (FS), disease specific survival rate, and overall survival rate compared with the rs738792 “CT + TT” carriers (Figure 1b–d). Previous study has suggested that the MMP11 may play a role as a predictor of poor survival and marker of metastasis in urothelial carcinomas [11]. Consistent with this result, the expressions of MMP11 rs131451 and rs738792 we discovered in our study may provide a possible link and mechanism to explain this phenomenon between the MMP11 SNPs and UCC prognosis (Figure 1). However, in contrast, it was suggested that the MMP11 rs738792 C allele was associated with higher risk to develop to Child-Pugh B or C grade in HCC patients [38], and the C allele of MMP11 rs738792 was also suggested to be associated with increased incidence of lymph node metastasis in OSCC patients [40]. Therefore, the reason why the MMP11 rs738792 “CC” genotype was associated with better UCC prognosis remained unclear, while no significant association was found between the rs738792 polymorphisms and clinical status of UCC (data not shown) and UCC susceptibility (Table 2). The limitation to our study is the lack of tissue or mRNA collection from our study cohort, so detailed analysis such as the impact between the difference in MMP11 genotypes and their protein expression to UCC progression could not be performed. Therefore, the exact mechanisms of the MMP11 SNPs in UCC carcinogenesis and regulations require future well-designed study to elucidate it.

## 5. Conclusions

In conclusion, our results demonstrated that the MMP11 SNPs are associated with UCC susceptibility, clinical status, disease progression, and survival. The MMP11 rs28382575, rs131451, and rs738792 polymorphisms may potentially provide as tumor markers in UCC treatment or predictors for UCC susceptibility and prognosis.

## Figures and Tables

**Table 1:** The distributions of demographical characteristics in 650 controls and 431 patients with UCC.

Variable	Controls (*N* = 650) *n* (%)	Patients (*N* = 431)*n* (%)	*p* Value
Age (yrs)			<0.001
≤65	423 (65.1%)	166 (38.5%)	
>65	227 (34.9%)	265 (61.5%)	
Mean ± S.D.	64.1 ± 3.1	68.6 ± 11.8	<0.001
Gender			0.991
Female	240 (36.9%)	159 (36.9%)	
Male	410 (63.1%)	272 (63.1%)	
Tobacco consumption			0.442
No	438 (67.4%)	300 (69.6%)	
Yes	212 (32.6%)	131 (30.4%)	
Stage			
Non muscle invasive tumor (pTa–pT1)		235 (54.5%)	
Muscle invasive tumor (pT2–pT4)		196 (45.5%)	
Tumor T status			
Ta		90 (20.9%)	
T1-T4		341 (79.1%)	
Lymph node status			
N0		380 (88.2%)	
N1 + N2		51 (11.8%)	
Metastasis			
M0		417 (96.8%)	
M1		14 (3.2%)	
Histopathologic grading			
Low grade		53 (12.3%)	
High grade		378 (87.7%)	

Student’s t test or Chi-squared test was used between controls and patients with UCC.

**Table 2:** Genotype Distributions of MMP11 Gene Polymorphisms in 650 Controls and 431 Patients with UCC.

Variable	Controls (*N* = 650) *n* (%)	Patients (*N* = 431) *n* (%)	OR (95% CI)	AOR (95% CI)
**rs738791**				
CC	309 (47.5%)	181 (42%)	1.000 (reference)	1.000 (reference)
CT	270 (41.5%)	203 (47.1%)	1.284 (0.991–1.662)	1.230 (0.939–1.610)
TT	71 (10.9%)	47 (10.9%)	1.130 (0.749–1.706)	1.192 (0.775–1.832)
CT + TT	341 (52.5%)	250 (58%)	1.252 (0.979–1.600)	1.222 (0.946–1.579)
**rs2267029**				
GG	365 (56.2%)	230 (53.4%)	1.000 (reference)	1.000 (reference)
AG	239 (36.8%)	172 (39.9%)	1.142 (0.884–1.476)	1.126 (0.862–1.47)
AA	46 (7.1%)	29 (6.7%)	1.000 (0.611–1.638)	1.009 (0.605–1.684)
AG + AA	285 (43.8%)	201 (46.6%)	1.119 (0.876–1.429)	1.107 (0.858–1.428)
**rs738792**				
TT	341 (52.5%)	207 (48%)	1.000 (reference)	1.000 (reference)
CT	253 (38.9%)	185 (42.9%)	1.205 (0.932–1.557)	1.169 (0.895–1.527)
CC	56 (8.6%)	39 (9%)	1.147 (0.736–1.788)	1.117 (0.704–1.773)
CT + CC	309 (47.5%)	224 (52%)	1.194 (0.936–1.524)	1.160 (0.900–1.495)
**rs28382575**				
TT	631 (97.1%)	405 (94%)	1.000 (reference)	1.000 (reference)
CT	19 (2.9%)	26 (6%)	2.132 (1.165–3.903)	2.045 (1.088–3.843)
CC	0 (0%)	0 (0%)	NA	NA
CT + CC	19 (2.9%)	26 (6%)	2.132 (1.165–3.903)	2.045 (1.088–3.843)
**rs131451**				
TT	233 (35.8%)	139 (32.3%)	1.000 (reference)	1.000 (reference)
CT	301 (46.3%)	215 (49.9%)	1.197 (0.911–1.574)	1.115 (0.839–1.482)
CC	116 (17.8%)	77 (17.9%)	1.113 (0.779–1.589)	1.136 (0.784–1.645)
CT + CC	417 (64.2%)	292 (67.7%)	1.174 (0.907–1.519)	1.121 (0.857–1.465)

Bold font indicates statistical significance (*p* < 0.05); NA: not applicable. The odds ratio (OR) with their 95% confidence intervals were estimated by logistic regression models. The adjusted odds ratio (AOR) with their 95% confidence intervals were estimated by multiple logistic regression models after controlling for age, gender and tobacco consumption.

**Table 3:** Distribution frequency of the clinical status and MMP11 (rs131451) genotype frequencies in 431 UCC patients.

	MMP11 (rs131451)			
Variable	CT + TT (%) (*n* = 354)	CC (%) (*n* = 77)	OR (95% CI)	*p* Value
**Stage**				
Non muscle invasive tumor (pTa–pT1)	189 (53.4%)	46 (59.7%)	1.000 (reference)	
Muscle invasive tumor (pT2–pT4)	165 (46.6%)	31 (40.3%)	0.772 (0.468–1.274)	0.311
**Tumor T status**				
Ta	69 (19.5%)	21 (27.3%)	1.000 (reference)	
T1-T4	285 (80.5%)	56 (72.7%)	0.646 (0.366–1.137)	0.130
**Lymph node status**				
N0	314 (88.7%)	66 (85.7%)	1.000 (reference)	
N1 + N2	40 (11.3%)	11 (14.3%)	1.308 (0.638–2.683)	0.463
**Metastasis**				
M0	344 (97.2%)	73 (94.8%)	1.000 (reference)	
M1	10 (2.8%)	4 (5.2%)	1.885 (0.575–6.176)	0.295
**Histopathologic grading**				
Low grade	42 (11.9%)	11 (14.3%)	1.000 (reference)	
High grade	312 (88.1%)	66 (85.7%)	0.808 (0.395–1.651)	0.558

Bold font indicates statistical significance (*p* < 0.05). The odds ratio (OR) with their 95% confidence intervals were estimated by logistic regression models.

**Table 4:** Distribution frequency of the clinical status and MMP11 (rs131451) genotype frequencies in 166 UCC patients among age ≤65 years old.

	MMP11 (rs131451)			
Variable	CT + TT (%) (*n* = 134)	CC (%) (*n* = 32)	OR (95% CI)	*p* Value
**Stage**				
Non muscle invasive tumor (pTa–pT1)	69 (51.5%)	19 (59.4%)	1.000 (reference)	
Muscle invasive tumor (pT2–pT4)	65 (48.5%)	13 (40.6%)	0.726 (0.332–1.589)	0.423
**Tumor T status**				
Ta	22 (16.4%)	11 (34.4%)	1.000 (reference)	
T1-T4	112 (83.6%)	21 (65.6%)	0.375 (0.159–0.887)	0.026
**Lymph node status**				
N0	112 (83.6%)	27 (84.4%)	1.000 (reference)	
N1 + N2	22 (16.4%)	5 (15.6%)	0.943 (0.327–2.716)	0.913
**Metastasis**				
M0	130 (97%)	29 (90.6%)	1.000 (reference)	
M1	4 (3%)	3 (9.4%)	3.362 (0.714–15.842)	0.125
**Histopathologic grading**				
Low grade	17 (12.7%)	6 (18.8%)	1.000 (reference)	
High grade	117 (87.3%)	26 (81.3%)	0.630 (0.226–1.752)	0.375

Bold font indicates statistical significance (*p* < 0.05). The odds ratio (OR) with their 95% confidence intervals were estimated by logistic regression models.

**Table 5:** Risk of death on genotypic distributions of *MMP11* gene polymorphisms (rs131451, rs738792) among 430 UCC patients.

Variable	N	Number of Death	HR (95% CI)	AHR (95% CI)
**Stage**				
Non muscle invasive tumor (pTa–pT1)	235	39	1.000 (reference)	1.000 (reference)
Muscle invasive tumor (pT2–pT4)	195	72	3.297 (2.222–4.893)	3.375 (2.272–5.015)
**Tumor T status**				
Ta	90	14	1.000 (reference)	1.000 (reference)
T1-T4	340	97	2.681 (1.524–4.716)	2.661 (1.51–4.69)
**Lymph node status**				
N0	379	85	1.000 (reference)	1.000 (reference)
N1 + N2	51	26	4.408 (2.803–6.934)	4.898 (3.105–7.726)
**Metastasis**				
M0	416	103	1.000 (reference)	1.000 (reference)
M1	14	8	6.561 (3.138–13.717)	8.566 (3.985–18.41)
**Histopathologic grading**				
Low grade	53	6	1.000 (reference)	1.000 (reference)
High grade	377	105	3.569 (1.56–8.164)	3.431 (1.497–7.863)
**rs131451**				
CC	77	13	1.000 (reference)	1.000 (reference)
CT + TT	353	98	2.089 (1.145–3.81)	2.05 (1.12–3.754)
**rs738792**				
CC	39	2	1.000 (reference)	1.000 (reference)
CT + TT	391	109	6.468 (1.593–26.258)	6.562 (1.612–26.704)

The hazard ratio (HR) with their 95% confidence intervals was estimated by Cox proportional hazards model. The adjusted hazard ratio (AHR) with their 95% confidence intervals were estimated by multiple Cox proportional hazards model after controlling for age, gender and tobacco consumption.
